# Fabrication and Characterization of Collagen/PVA Dual-Layer Membranes for Periodontal Bone Regeneration

**DOI:** 10.3389/fbioe.2021.630977

**Published:** 2021-06-09

**Authors:** Tian Zhou, Siwei Chen, Xinxin Ding, Zhihuan Hu, Lian Cen, Xiaomeng Zhang

**Affiliations:** ^1^Department of Oral Maxillofacial-Head and Neck Oncology, Shanghai Ninth People’s Hospital, College of Stomatology, Shanghai Jiao Tong University School of Medicine, Shanghai, China; ^2^National Clinical Research Center for Oral Diseases, Shanghai Key Laboratory of Stomatology and Shanghai Research Institute of Stomatology, Shanghai, China; ^3^Shanghai Key Laboratory of Multiphase Materials Chemical Engineering, State Key Laboratory of Chemical Engineering, Department of Product Engineering, School of Chemical Engineering, East China University of Science and Technology, Shanghai, China; ^4^Department of Implant Dentistry, Shanghai Ninth Peoples’ Hospital, College of Stomatology, Shanghai Jiao Tong University School of Medicine, Shanghai, China

**Keywords:** dual-layer, periodontal, collagen, PVA, GTR

## Abstract

Guided tissue regeneration (GTR) is a promising treatment for periodontal tissue defects, which generally uses a membrane to build a mechanical barrier from the gingival epithelium and hold space for the periodontal regeneration especially the tooth-supporting bone. However, existing membranes possess insufficient mechanical properties and limited bioactivity for periodontal bone regenerate. Herein, fish collagen and polyvinyl alcohol (Col/PVA) dual-layer membrane were developed via a combined freezing/thawing and layer coating method. This dual-layer membrane had a clear but contact boundary line between collagen and PVA layers, which were both hydrophilic. The dual membrane had an elongation at break of 193 ± 27% and would undergo an *in vitro* degradation duration of more than 17 days. Further cell experiments showed that compared with the PVA layer, the collagen layer not only presented good cytocompatibility with rat bone marrow-derived mesenchymal stem cells (BMSCs), but also promoted the osteogenic genes (RUNX2, ALP, OCN, and COL1) and protein (ALP) expression of BMSCs. Hence, the currently developed dual-layer membranes could be used as a stable barrier with a stable degradation rate and selectively favor the bone tissue to repopulate the periodontal defect. The membranes could meet the challenges encountered by GTR for superior defect repair, demonstrating great potential in clinical applications.

## Introduction

Periodontitis is an infectious disease that causes the detachment of the junctional epithelium from the tooth layer and the disconnection of periodontal ligament fibers, and eventually leads to bone loss (Xu et al., 2012). Guided tissue regeneration (GTR) is an effective treatment to control disease progression and promote the formation of tooth-supporting tissues. Ideal GTR membranes are expected to act as barriers that can prevent the migration of epithelial cells into the defect areas while hold space to guide the regeneration of impaired tissues including cementum, periodontal ligament, and alveolar bone (Liu X. et al., 2020). Collagen membranes, as conventional bioabsorbable membranes, are mostly used for GTR in clinical practice. However, they degrade and collapse quickly, followed by epithelial ingrowth and compromised bone formation. In addition, collagen membranes are mostly chemically crosslinked and lack osteogenic potential. Therefore, it would be a win–win solution to develop a design of a novel dual-layer GTR membrane, of which one layer could prevent epithelium ingrowth and the other layer could promote selective repopulation of the root surface by multipotent cells (i.e., periodontal ligament cells and osteoblasts).

Polyvinyl alcohol (PVA) is a water-soluble polymer with excellent biocompatibility and easy to process for biomedical applications (Chaudhuri et al., 2016). With tissue-like elasticity and excellent mechanical strength, PVA can be used to improve the mechanical properties of other materials. Lin et al. (2020) have reported that the tensile and compressive properties of bioactive glass crosslinked PVA hydrogel were enhanced with the increase in PVA content. PVA membrane also can act as a physical barrier to prevent adhesion after surgery (Weis et al., 2004). The viability of human gingival fibroblasts (HGFs) was decreased by increasing the ratio of PVA in PVA/collagen composite hydrogels (Zhou et al., 2020), which showed the potential of PVA hydrogel for resisting gingival tissue ingrowth. Hence, PVA was chosen for acting as the outer layer of the current novel GTR membrane for providing mechanical support and preventing the fast attachment of epithelium.

Regarding the inner layer, collagen has been widely used in periodontal tissue regeneration for its excellent biocompatibility, biodegradability, and low antigenicity (Jiang et al., 2017). However, currently available collagen is mainly extracted from mammals such as bovine and porcine, which has religious limitation on Jews, Muslims, and Hindus (Goyal et al., 2013), and also has the risk of the prevalence of transmitting animal diseases (Chuaychan et al., 2015). In recent years, marine collagen has gained increasing attention as a safer alternative source of mammalian collagen (Paradiso et al., 2019). Song et al. (2006) have reported that jellyfish collagen exhibited higher fibroblast viability than other naturally derived biomaterials, including bovine collagen, gelatin, hyaluronic acid, and glucan. Tilapia collagen exhibited the effect of promoting osteogenesis and fibroblast formation and is also beneficial to the proliferation of human vascular endothelial cells (Song et al., 2019). According to these reports, fish collagen might have great potential to be used as the inner layer of the current design of GTR membrane to improve the bioactivity of multipotential cells. Although the inner layer made of collagen has relatively poor mechanical property and rapid degradation rate (Raftery et al., 2016), the outer layer made of PVA could make up for that. However, it is still unknown whether the dual-layer membrane could be tailored by taking advantage of fish collagen and PVA. Besides, whether the mechanical property and the osteogenic bioactivity of this novel dual-layer membrane can be improved as expected also needs to be verified.

Therefore, the main aim of this study was to develop a collagen (Col)/PVA dual-layer membrane and investigate its potential for promoting the desired regeneration of impaired tissue within periodontal defect areas. The PVA layer was first partially pre-set according to a freezing/thawing method, while the collagen layer was initiated by layer coating onto the partially set PVA layer without any chemical crosslinking involved. Cross-sectional morphology of the dual-layer membrane was observed by SEM, while its mechanical strength was represented by tensile strength. *In vitro* degradation performance was further determined. Adhesion and proliferation of rat bone marrow mesenchymal stem cells (BMSCs) on each layer of the membrane, as well as osteogenic-related genes, such as runtrelated gene-2 (RUNX2), alkaline phosphatase (ALP), osteocalcin (OCN), and collagen 1 (COL1) and protein (ALP) expression by the attached BMSCs were assayed with time. This study could provide substantial demonstration to realize such a design and also to verify the feasibility of this dual-layer Col/PVA membrane as a novel GTR membrane, and the controllable methodology also exhibits great potential in clinical applications.

## Materials and Methods

### Preparation of Col/PVA Dual-Layer Membrane

Tilapia scale collagen was provided by Shanghai Fisheries Research Institute. It was first dissolved in 0.1 M acetic acid at room temperature with stirring for 4 h to form a 1.5% collagen solution. At the same time, PVA (AR 1799, General-Reagent) was dissolved in water at 80^*o*^C with magnetic stirring for 6 h to prepare an 18% PVA solution. The dispersion of PVA solution of high viscosity required a varying stirring speed of around 150–250 rpm. After that, PVA solution was spread evenly in a petri dish of 60 mm-diameter and placed at room temperature for 24 h to crosslink. The PVA membrane was then pre-frozen at −20^*o*^C for 1 h and the above-mentioned collagen solution was spread evenly on the pre-frozen membrane layer. The mixture was then placed at −20^*o*^C for 24 h. Finally, the dual-layer membrane was immersed in 0.1 M NaOH solution for 10 s and then rinsed with adequate DI water.

### Characterization of Col/PVA Dual-Layer Membrane

The morphology of the Col/PVA dual-layer membrane was observed with a scanning electron microscope (SEM) (Nova SEM 450, FEI, United States) with the platinum spray time of 90 s and the voltage of 10 kV. The weight loss temperature of the dual-layer membrane was determined by a thermogravimetric (TG) analyser (Q500, TA, United States). The membrane sample was heated at the rate of 10^*o*^C/min in a temperature range of 30–800^*o*^C. The characterization tests for pure collagen membrane and PVA membrane were also conducted as comparison. The wettability data of both sides of the dual-layer membrane was obtained by a contact angle measuring instrument (OCA20, Dataphysics, Germany). To be specific, 5 μl of water droplets were used for water contact angle (WCA) measurement.

### Mechanical Strength

The mechanical strength of the membrane was measured by a universal testing machine (Instron 5542, United States) with a stretching speed of 20 mm/min. The membrane was cut into a dimension of 10 mm × 30 mm for the test. The mechanical test is conducted on wet samples which were pre-immersed in DI water for 30 s. The sample thickness was observed through a microscope (BX51-P, Olympus, Japan). In order to prevent the test sample from sliding or falling off the clamps during stretching processes, medical gauze is added between the sample and the clamp to increase friction. Elongation at break was calculated as (*L*_*b*_–*L*_*o*_)/*L*_*o*_, where *L*_*b*_ is the length of samples when the tensile force reaches zero, which means the sample is totally broken, and *L*_*o*_ is the length of an exposed sample between two test clamps before stretching. *L*_*o*_ was measured by a vernier caliper and *L*_*b*_ was determined by the universal testing machine. Tensile strength was calculated as *F*_*max*_/*A*_*o*_, where *F*_*max*_ is the maximum of tensile force during stretching and *A*_*o*_ is the cross-section area of the sample before stretching.

### *In vitro* Degradation Test and Morphology After Immersion in PBS

The membranes were irradiated under ultraviolet light for 12 h to kill bacteria, and all operations were performed on a sterile operating table (Hamanaka et al., 2011). Each membrane was cut into 10 mm × 30 mm samples. Each sample was immersed in 5 ml PBS solution at 37^*o*^C for different time durations. The PBS solution was replaced every 4 days. After predetermined time durations, the test samples were withdrawn and lyophilized by a freeze dryer. The remaining weight of each sample was measured. The degradation level was calculated in weight % using the following equation:

$$\text{Degradation}\,\left[w\,t\%\right]=\frac{\text{Mc}}{\text{Mi}}\times 100$$

in which Mc is the weight at the time of measurement, and Mi corresponds to the initial mass of the sample prior to immersion in PBS. The Cross-section morphology of lyophilized Col/PVA dual-layer membranes after immersion in PBS for 4, 8, 12, and 17 days was also observed by SEM.

### Rat BMSCs Culture

Rat BMSCs were obtained from the 4-week-old Sprague-Dawley rats. Approval for bone marrow harvesting was obtained from the Animal Care and Experiment Committee of Ninth People’s Hospital affiliated to School of Medicine, Shanghai Jiao Tong University. Rat BMSCs were isolated and harvested as described in the literature (Liu and Sun, 2014). Under aseptic conditions, bilateral tibiae and femora were dissected from the muscle and connective tissue, and the epiphyses were removed. Cells were cultured in Dulbecco’s modified eagle medium (DMEM, Hyclone) with10% fetal bovine serum (FBS, Hyclone), 100 U/mL penicillin, and 100 mg/L streptomycin (Hyclone) and incubated at 37°C with 5% CO_2_ for 24 h. The BMSCs were used for the experiments in passage 2–3.

### Cell Viability and Proliferation Assay

BMSCs viability and proliferation were evaluated by the Cell Counting Kit-8 (CCK-8, Dojindo, Japan) assay. Briefly, BMSCs were seeded, respectively, on the collagen layer of the Col/PVA dual-layer membrane and pure PVA membrane in 96-well plates at a cell density of 5 × 10^3^ cells per well for 1, 3, and 5 days. The optical density (OD) of formazan at 450 nm was measured using a Multiskan GO microplate photometer (Thermo Scientific, United States). At 1 day, hochest and EdU staining (Beyotime, Shanghai, China) was performed to detect the DNA duplicate activity and the cells were examined collagen using the fluorescence microscope.

### Osteogenic Genes Expression

Expression of marker genes of osteoblasts, such as RUNX2, ALP, OCN, and COL1 was detected by real-time polymerase chain reaction (PCR), and the housekeeping gene GAPDH was used to normalize results. In brief, BMSCs were seeded, respectively, on the layer of the Col/PVA dual-layer membrane and pure PVA membrane in 6- well plates at a cell density of 1 × 10^5^ cells per well for 7 and 14 days. Total RNA extraction was isolated using TRIZOL (Invitrogen, United States) according to the manufacturer’s instructions. Gene expression level was tested by a Bio-Rad sequence detection system (MyiQ2, United States) using a real-time PCR kit (SYBR Premix EX Taq, TaKaRa). The sequences of specific primers for these genes are listed in Table 1.

**TABLE 1:** Primer sequences used for qRT-PCR.

**Gene**	**Forward sequence**	**Reverse sequence**
RUNX2	TCCGCCACCACTCACTACCAC	GGAACTGATAGGACGCTGACGAAG
ALP	GCCTACTTGTGTGGCGTGAA	AGGATGGACGTGACCTCGTT
OCN	GGACCCTCTCTCTGCTCACTCTG	ACCTTACTGCCCTCCTGCTTGG
COL1	CGAGTCACACCGGAACTTGG	CCAATGTCCAAGGGAGCCAC
GAPDH	CTGAACGGGAAGCTCACTGG	TGAGGTCCACCACCCTGTTG

### ALP Staining and Activity Assay

ALP staining was performed using a BCIP/NBT ALP kit (Beyotime, Shanghai, China). Briefly, BMSCs were seeded, respectively, on the collagen layer of the Col/PVA dual-layer membrane and pure PVA membrane in 6- well plates at a cell density of 1 × 10^5^ cells per well for 3 days. After washed by PBS, the BMSCs were fixed by 4% paraformaldehyde for 30 min, and were dyed in a mixture of nitro-blue tetrazolium and 5-bromo-4-chloro-3-indolylphosphate (Zhang et al., 2015). Then the cells were observed and photographed by optical microscopy. ALP activity on day 7 was also assessed using ALP detection kit (Beyotime, Shanghai, China) according to the manufacturer’s instructions. The ALP activity was normalized to the total protein content measured by BCA method.

### Statistical Analysis

Data are expressed as mean ± standard deviation (SD). All statistical analyses were performed using SPSS 20.0 software. Statistically significant differences (*p* < 0.05) between the two groups were evaluated using student’s t-test. Data analysis involved the use of Graph Pad Prism 8.2.1 software.

## Results and Discussion

### Characterization of the Col/PVA Dual-Layer Membrane

**FIGURE 1:**
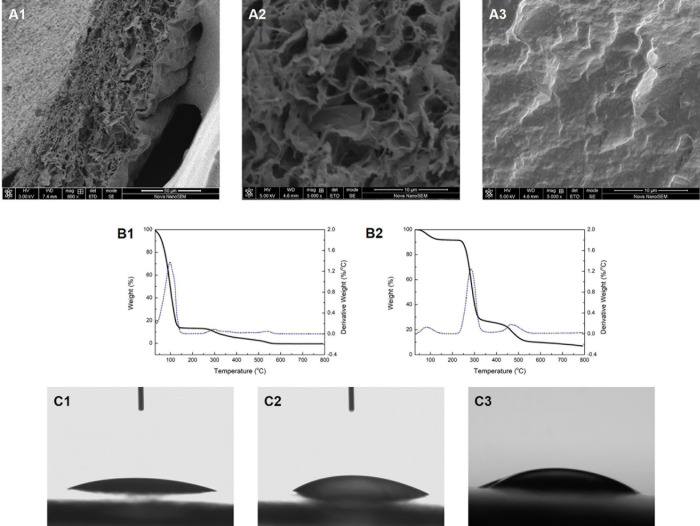
The cross-section morphology of the Col/PVA dual-layer membrane showing **(A1)** both layers, **(A2)** collagen layer, **(A3)** PVA layer. TG analysis of **(B1)** Col/PVA dual-layer membrane, **(B2)** pure PVA membrane. WCA of **(C1)** collagen side, **(C2)** PVA side of the Col/PVA dual-layer membrane, **(C3)** pure PVA membrane.

SEM images were taken to reveal the morphology of the membrane. Figure 1. A1 shows the cross-section of the Col/PVA membrane with a clear dual-layer structure and close connection at the interface. It was shown that the gelation of PVA dissolved in water upon the freezing/thawing cycle is due to partial crystallization of PVA chains that results in hard crystallites serving as physical cross-links of a network structure (Zhang et al., 2012). Moreover, a certain amount of free hydroxyl groups of PVA and enough chain mobility is likely to contributing to the formation of hydrogen bonding between PVA chains. The collagen solution was spread evenly on the pre-frozen PVA membrane layer which was partially set or gelated. It was thus proposed that free hydroxyl groups on PVA chains could lead to the formation of hydrogen bonding across the interface of PVA layer and collagen layer, since extensive hydrogen bonding formation could be expected between PVA chains when the two cut surfaces of PVA are brought into contact. The thickness of each layer could be easily adjusted by changing the amount of PVA solution and collagen solution added to the petri dish. Furthermore, it is obvious that each layer exhibits different morphology. The collagen layer has a loose and porous structure (Figure 1. A2), while PVA layer has a dense and highly crosslinked structure (Figure 1. A3). The porous network structure of collagen could facilitate new-born cells to migrate into the scaffold and guide cells to proliferate. The size and porosity of the collagen materials can be effectively controlled by adjusting the freeze-drying rate, where large pore size can permit cell migration and nutrient diffusion and small pore sizes can promote cell adhesion (Sorushanova et al., 2019). However, extremely small pores should also be avoided as cells cannot migrate toward the center of the construct by limiting the diffusion of nutrients and removal of waste products (Murphy and O’Brien, 2010), thus further restricting cell attachment and differentiation potential. In our study, the collagen layer exhibits a microporous structure, which was supposed to permit cell ingrowth. Meanwhile, PVA would act as a barrier with a dense structure that could inhibit other cells from invading the collagen layer. In this way, PVA layer holds adequate space for new-born cells to grow. These properties could be further manipulated with the adjustment of the relative thickness of each layer to achieve the desired biological responses.

The TG analysis and the first derivative to the rate of weight loss are shown in Figures 1. B1,B2. The weight loss of the Col/PVA double-layer membrane mainly occurred at 30–147 and 260–575^*o*^C (Figure 1. B1). The first stage of weight loss was mainly due to the loss of moisture and residual acetic acid (Lewandowska et al., 2016). The second stage might be attributed to the thermal degradation of collagen and PVA. The weight loss of pure collagen solution occurred at 30–163^*o*^C, during which the rate of thermal weight loss continued to rise. PVA membrane lost weight at 50–130, 230–340, and 440–540^*o*^C, respectively, as shown in Figure 1. B2. These three major stages were, respectively, referred to the weakly physisorption of water, the decomposition of PVA side chain, and the decomposition of PVA main chain (Asran et al., 2010). TG analyze indicated that the weight loss behavior of Col/PVA dual-layer membrane was regulated by the properties of collagen and PVA and their relative contents. With the combination of PVA, the weight loss rate of the dual-layer membrane was decreased compared with that of the pure collagen membrane. Therefore, the Col/PVA dual-layer membrane showed thermodynamically stability for application in the human body.

The WCA of both sides of the Col/PVA dual-layer membrane was shown in Figures 1. C1,C2. The collagen layer had a WCA of 20^*o*^ ± 1^*o*^ (Figure 1. C1), and PVA layer had a WCA of 30^*o*^ ± 1^*o*^ (Figure 1. C2). Pure PVA membrane had a WCA of 33^*o*^ ± 1^*o*^ (Figure 1. C3). It indicated that both layers have great hydrophilic performance. Hence, the inherent excellent hydrophilicity of both collagen and PVA was preserved. It is known that collagen with amino acid contents is highly hydrophilic and can initiate the release of biological signals to promote cell adhesion and proliferation (Sobhanian et al., 2019). PVA also has good hydrophilicity and is highly soluble in water and environmentally safe, suitable as a component for membrane modification and property enhancement (Park et al., 2018). The manufacturing process of the dual-layer Col/PVA membrane is a purely physical process, which retains both the physical and chemical properties of collagen and PVA material in each layer, showing superior hydrophilicity as a desirable feature of GTR membranes.

### Mechanical Properties of the Col/PVA Dual-Layer Membrane

**FIGURE 2:**
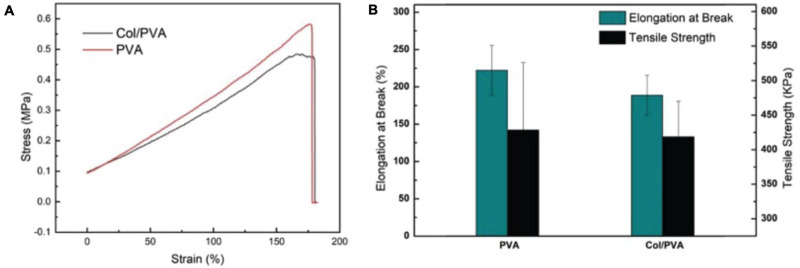
Mechanical properties of the Col/PVA dual-layer membrane and pure PVA membrane **(A)** tensile stress–strain curve **(B)** Elongation at break and tensile strength.

The mechanical strength of the Col/PVA dual-layer membrane and the pure PVA membrane were compared in Figure 2. The PVA membrane has an excellent elasticity with the elongation at break of 222 ± 33% and the tensile strength of 429 ± 98 KPa. After the combination of collagen layer, the Col/PVA dual-layer membrane preserved the close mechanical strength to that of pure PVA membrane. It has an elongation at break of 193 ± 27% and the tensile strength of 419 ± 51 KPa. However, there is no significant difference between the two groups, indicating that the desired mechanical strength from PVA is highly reserved after the addition of collagen layer under the current design and processing conditions.

Guided tissue regeneration membranes need to endure the tensile force from tissue extraction and possess high flexibility to keep structural integrity during the surgical process (Geão et al., 2019). Pure collagen membrane of this our thin-layer scale is too mechanically fragile to test its tensile strength, since it would break when it was placed between testing clamps. PVA is a flexible polymer and can be used to improve the properties of polymers (Aadil et al., 2018). It is robust and has a high strength-to-weight ratio (Chaudhuri et al., 2016). It has been reported that in the process of fabricating collagen/PVA composites patches, the mechanical strength could be increased by increasing the concentration of PVA with a constant collagen amount (Iqbal et al., 2018). Therefore, in our study, adding a PVA layer on the pure collagen membrane is expected to bring adequate mechanical strength. The results showed that the Col/PVA dual-layer membrane is highly flexible with a high elongation, which not only makes the Col/PVA dual-layer membrane easy to operate in clinic, but also provides mechanical support for bone regeneration.

### *In vitro* Degradation of the Col/PVA Dual-Layer Membrane

**FIGURE 3:**
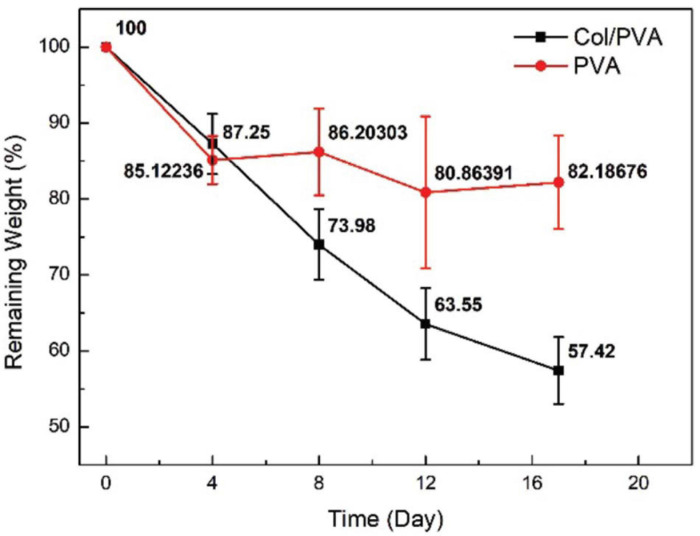
Remaining weight of Col/PVA and PVA membrane after being immersed in PBS for different time durations.

The degradation of the Col/PVA dual-layer membranes was evaluated, and it was shown that the weight of the membrane was decreasing when it was immersed in PBS solution as shown in Figure 3. The weight of samples decreased with time almost linearly for ∼12 days. The first 4 days had a weight drop of 12.7 ± 4.8%(*p* < 0.05, day 0 versus 4), while the second 4 days had a weight drop of 13.3 ± 5.7% (*p* < 0.05, day 4 versus 8) and the third 4 days was 10.4 ± 5.8%(*p* < 0.1, day 8 versus 12). From day 12 on, the degradation was slowed down and the remaining weight was maintained around 58–67% after day 17. The degradation performance of pure PVA membrane was also evaluated, and the remaining weight was maintained around 80–86%. In this study, PVA was used to improve the degradation property of collagen, just as Rebia et al. (2018) reported that the degradation rate of poly (3-hydroxybutyrate-co-3-hydroxyhexanoate) (PHBH) /PVA blend nanofibers could be greatly improved with the increased ratio of PVA polymer.

**FIGURE 4:**
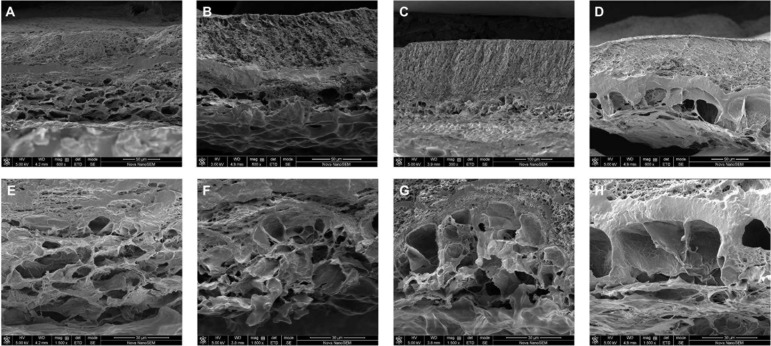
Cross-section morphology of lyophilized Col/PVA dual-layer membranes after being immersed in PBS for **(A)** 4 days, **(B)** 8 days, **(C)** 12 days, **(D)** 17 days, and the corresponding magnified images of collagen layer at **(E)** 4 days, **(F)** 8 days, **(G)** 12 days, **(H)** 17 days.

The morphology of the membrane immersed in PBS at different time points was further observed by SEM. From Figures 4A–D, it was obvious that PVA layer remained a dense structure, indicating that PVA degraded slowly and can be a reliable barrier. Meanwhile, the collagen layer exhibited desirable changes to a porous structure. From the images shown in Figures 4E–H, the sheet-like porous network of collagen layer was gradually changed with the increase in the pore size and the appearance of local collapse of the microstructure (Figure 4H). The increased pore size would be beneficial for new-born cells to grow because the cells need more space to proliferate and spread to the inside of the collagen scaffold. It is normally identified that pore sizes in the 10–150 μm range in scaffolds intended as support for growing cells (Montalbano et al., 2018). Our results showed that the Col/PVA dual-layer membrane could endure immersion in PBS for at least 17 days and kept the desirable porous size on the collagen side, which was advantageous to induce the bone-forming activity of osteoblastic cells.

### Viability and Proliferation of BMSCs Cultured With Col/PVA Dual-Layer Membrane

**FIGURE 5:**
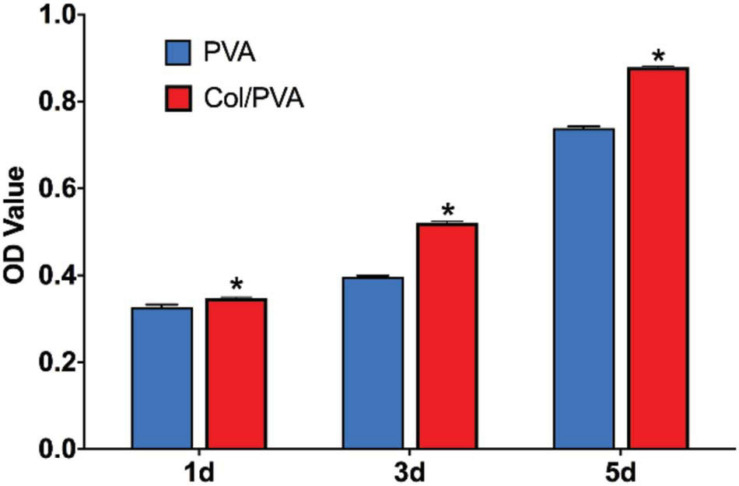
CCK-8 assay results of BMSCs cultured for 1, 3, and 5 days. Data are presented as mean values with standard deviation. *indicates a significant difference between Col/PVA and PVA group with *p*-value < 0.05.

**FIGURE 6:**
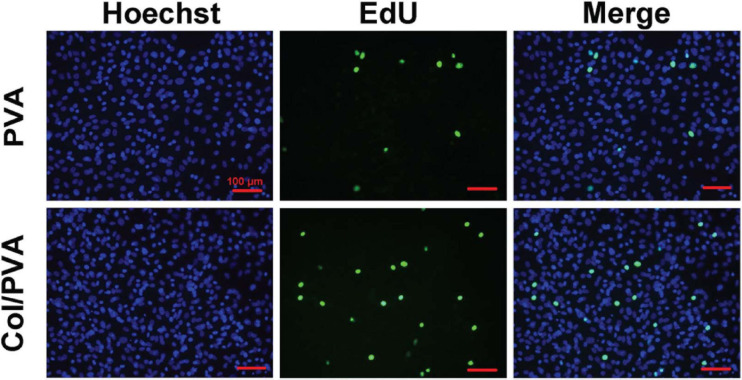
Fluorescence images of BMSCs cultured on the pure PVA membrane and collagen layer of the Col/PVA dual-layer membrane (represented as Col/PVA) for 1 day. Hoechst staining with blue indicated the living cells on two surfaces and EdU staining with green indicated the active DNA duplicate activity of cells. The scale bar was 100 μm.

Figure 5 shows the viability of BMSCs cultured on the collagen layer of the Col/PVA dual-layer membrane and pure PVA membrane at 1, 3, and 5 days. Compared to the pure PVA membrane, the collagen layer of the Col/PVA dual-layer membrane had more viable BMSCs at 1, 3, and 5 days. There is a significant difference between the collagen layer and pure PVA membrane (*p* < 0.05). Figure 6 shows the Hoechst and EdU staining of the BMSCs at 1 day. It was obvious that the collagen layer of the Col/PVA dual-layer membrane had better cell proliferation activity than pure PVA membrane. The fluorescence microscope images were also conformed to the result of CCK-8 assay, indicating that there was a cause-and-effect relationship between the enhanced proliferation activity and cell viability of BMSCs induced by collagen. In our study, the collagen layer used as the inner side of the Col/PVA dual-layer membrane was intended for placing in the bone side of a GTR-treated defect. Therefore, it is advantageous for osteoblastic cells to populate the collagen layer. The results exhibited the improved cell proliferation of BMSCs on the collagen layer, just as many reports also proved that bone tissue-derived cells are able to attach to and to proliferate on different collagen membranes *in vitro* (Behring et al., 2008). The good biocompatibility of the collagen layer was associated with its porous structures and hydrophilicity. Zhang et al. (2017) reported that with the combination of the collagen, poly(L-lactide-co-glycolide)/β-tricalcium phosphate (PLGA/TCP) -collagen materials had the perforative microporous structures and improved hydrophilicity for supporting cell seeding and promoting cell adhesion. Figures 1, 4 showed that the collagen layer had a large number of micropores, which can effectively improve the specific surface area of scaffolds to enable their supporting nutrient and oxygen transport during bone regeneration, and to ensure adequate hydrophilicity to allow cells to grow into the scaffolds.

### Differentiation of BMSCs Cultured With Col/PVA Dual-Layer Membrane

**FIGURE 7:**
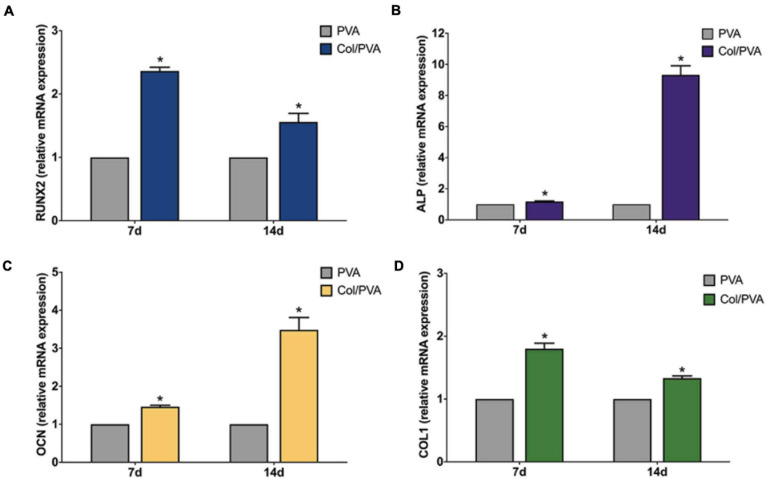
Expression profile of osteogenic differentiation related genes **(A)** RUNX2, **(B)** ALP, **(C)** OCN, **(D)** COL1 of BMSCs cultured on the collagen layer of the Col/PVA dual-layer membrane and pure PVA membrane for 7 and 14 days. *indicates a significant difference between Col/PVA and PVA group with *p*-value < 0.05.

Figure 7 shows the gene expression analysis of BMSCs cultured on the collagen layer of the Col/PVA dual-layer membrane and pure PVA membrane for 7 and 14 days. The osteoblastic gene expression of RUNX2, ALP, OCN, and COL1 were significantly upregulated on the collagen layer of the Col/PVA dual-layer membrane at 7 and 14 days as compared to that of the PVA layer (*P* < 0.05). The results demonstrating the expression of osteogenic genes can be induced by the collagen layer of Col/PVA dual-layer membrane. RUNX2 is an essential transcription factor required for osteogenic differentiation. Elango et al. (2019) has reported that collagen peptide (CP) triggered p38MAPK dependent RUNX2 signaling pathway in BMMS cells during osteoblast differentiation. ALP is an early marker for osteogenic differentiation, which can provide the necessary phosphate groups for promoting the formation of bone-like nodules. OCN is a specific marker of mature osteoblasts and is synthesized by fully differentiated osteoblasts. Collagen has outstanding osteoconductive property. It was reported that coating collagen on PLGA fibers could enhance the expression of osteogenic marker genes (RUNX2, ALP, and OCN) of rMSCs (Yang et al., 2018). COL1 gene, a crucial element of connective tissue, is also a marker of osteogenic differentiation related to bone formation and bone architecture (Hayrapetyan et al., 2016). The data in the present study have confirmed that the RUNX2, ALP, OCN, and COL1 gene expression induced by the collagen layer of Col/PVA dual-layer membrane was significantly higher than that of the pure PVA membrane, which indicated that collagen layer facilitated osteoblastic differentiation of BMSCs and in turn can help to repair the alveolar during periodontal tissue regeneration.

### ALP Staining and Activity of BMSCs Cultured With Col/PVA Dual-Layer Membrane

**FIGURE 8:**
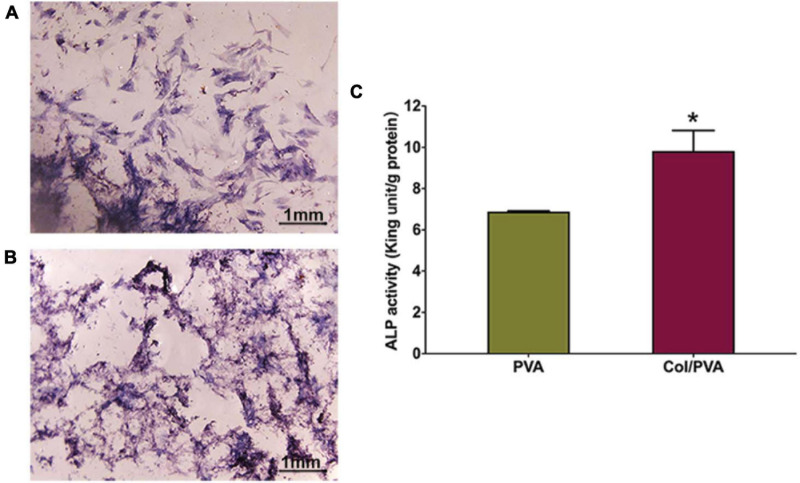
The ALP staining images of BMSCs cultured on the **(A)** pure PVA membrane and **(B)** the collagen layer of the Col/PVA dual-layer membrane for 7 days. **(C)** ALP activity of BMSCs cultured on the different surfaces for 7 days. *indicates a significant difference between PVA and Col/PVA group with *p*-value < 0.05.

ALP staining was used to detect the differences of the expression on the collagen layer of the Col/PVA dual-layer membrane and pure PVA membrane. As shown in Figures 8A,B, the BMSCs cultured on the collagen layer of the Col/PVA dual-layer membrane had enhanced ALP activity compared to the PVA membrane and the at 7 day (*P* < 0.05). ALP staining of BMSCs in Figure 8C also indicated that collagen layer of the Col/PVA dual-layer membrane displayed stronger ALP expression. These results indicate that the osteoblastic differentiation of BMSCs was stimulated by the collagen layer of the Col/PVA dual-layer membrane. Collagen is known to be an effective substrate for mineral deposition. Its layer contains sites that can promote osteoblast adsorption and mineral deposition. Volumes of new bone, new cementum and new periodontal ligament were significantly greater in the collagen hydrogel/sponge scaffold (Kosen et al., 2012). Collagen materials had osteogenic effects on human bone marrow-derived mesenchyme stem cells (Li et al., 2019). Montalbano et al. (2018). reported that ALP production in human mesenchymal stem cells increased at higher concentrations of collagen in collagen/alginate/fibrin based hydrogels, indicating their potential for the promotion of osteogenic activity. Moreover, collagen membrane when enriched with human periodontal-ligament stem cells and extracellular vesicles were shown to promote both bone regeneration and vascularization (Pizzicannella et al., 2019a), while bovine pericardium collagen membrane in together with human gingival MSCs and ascorbic acid could further perfect the osteogenic process (Pizzicannella et al., 2019b). In the renewal of bone tissue defects, a suitable scaffold plays a key role for proper bone formation and remodeling (Diomede et al., 2020). All of the above results thus fully substantiate the suitability of current Col/PVA membrane as a promising GTR for dental defects repair. In addition to the collagen based artificial scaffolds or membranes, it is worthy to mention another promising type of scaffold, based more on the extracellular matrix as well as cells, named as “cell sheet” (Chen et al., 2019; Lu et al., 2019). Cell sheet was efficaciously utilized in the regeneration of bone and periodontal ligament-like tissues as shown by recent works (Dong et al., 2020; Liu Y. et al., 2020). Promising as it demonstrated to be, cell sheet manufacture requires high manipulation skills to ensure the quality control (Chen et al., 2019), while the current artificial Col/PVA membrane offers the superiority in relatively easy manufacturing with controllability.

## Conclusion

The study developed a beneficial Col/PVA dual-layer membrane for periodontal guided bone regeneration. The characterization results suggested that the Col/PVA dual-layer membrane had a clear but contact boundary line between PVA and collagen layers with favorable hydrophilic property. Further research confirms that the collagen layer of the Col/PVA dual-layer membrane can not only enhance BMSCs viability but also induce the osteogenic differentiation of BMSCs by activating expression of RUNX2, ALP, OCN, and COL1. These findings opened up a new way for the application of Col/PVA dual-layer membrane in the field of periodontal regenerative therapy.

## Data Availability Statement

The raw data supporting the conclusions of this article will be made available by the authors, without undue reservation.

## Ethics Statement

The animal study was reviewed and approved by The Animal Care and Experiment Committee of Ninth People’s Hospital affiliated to School of Medicine, Shanghai Jiao Tong University.

## Author Contributions

TZ and LC contributed to the study conception and design. SC, XD, and ZH contributed to the fabrication of materials and study conduct. TZ and SC wrote the original manuscript. XZ and LC reviewed and edited the manuscript. XZ contributed to the analysis of data and supervision. All authors contributed to the article and approved the submitted version.

## Conflict of Interest

The authors declare that the research was conducted in the absence of any commercial or financial relationships that could be construed as a potential conflict of interest.
